# Cell-in-Cell Phenomenon and Its Relationship With Tumor Microenvironment and Tumor Progression: A Review

**DOI:** 10.3389/fcell.2019.00311

**Published:** 2019-12-03

**Authors:** Xinlong Wang, Yilong Li, Jiating Li, Le Li, Hong Zhu, Hua Chen, Rui Kong, Gang Wang, Yongwei Wang, Jisheng Hu, Bei Sun

**Affiliations:** ^1^Department of Pancreatic and Biliary Surgery, The First Affiliated Hospital of Harbin Medical University, Harbin, China; ^2^Key Laboratory of Hepatosplenic Surgery, Ministry of Education, The First Affiliated Hospital of Harbin Medical University, Harbin, China; ^3^Department of Pathology, The First Affiliated Hospital of Harbin Medical University, Harbin, China

**Keywords:** cell-in-cell, cannibalism, emperipolesis, entosis, tumor microenvironment, tumor progression

## Abstract

The term cell-in-cell, morphologically, refers to the presence of one cell within another. This phenomenon can occur in tumors but also among non-tumor cells. The cell-in-cell phenomenon was first observed 100 years ago, and it has since been found in a variety of tumor types. Recently, increasing attention has been paid to this phenomenon and the underlying mechanism has gradually been elucidated. There are three main related process: cannibalism, emperipolesis, and entosis. These processes are affected by many factors, including the tumor microenvironment, mitosis, and genetic factors. There is considerable evidence to suggest that the cell-in-cell phenomenon is associated with the prognosis of cancers, and it promotes tumor progression in most situations. Notably, in pancreatic cancer, the cell-in-cell phenomenon is associated with reduced metastasis, which is the opposite of what happens in other tumor types. Thus, it can also inhibit tumor progression. Studies show that cell-in-cell structure formation is affected by the tumor microenvironment, and that it may lead to changes in cellular characteristics. In this review, we summarize the different cell-in-cell processes and discuss their role in tumor progression and how they are regulated by different mechanisms.

## Introduction

**FIGURE 1:**
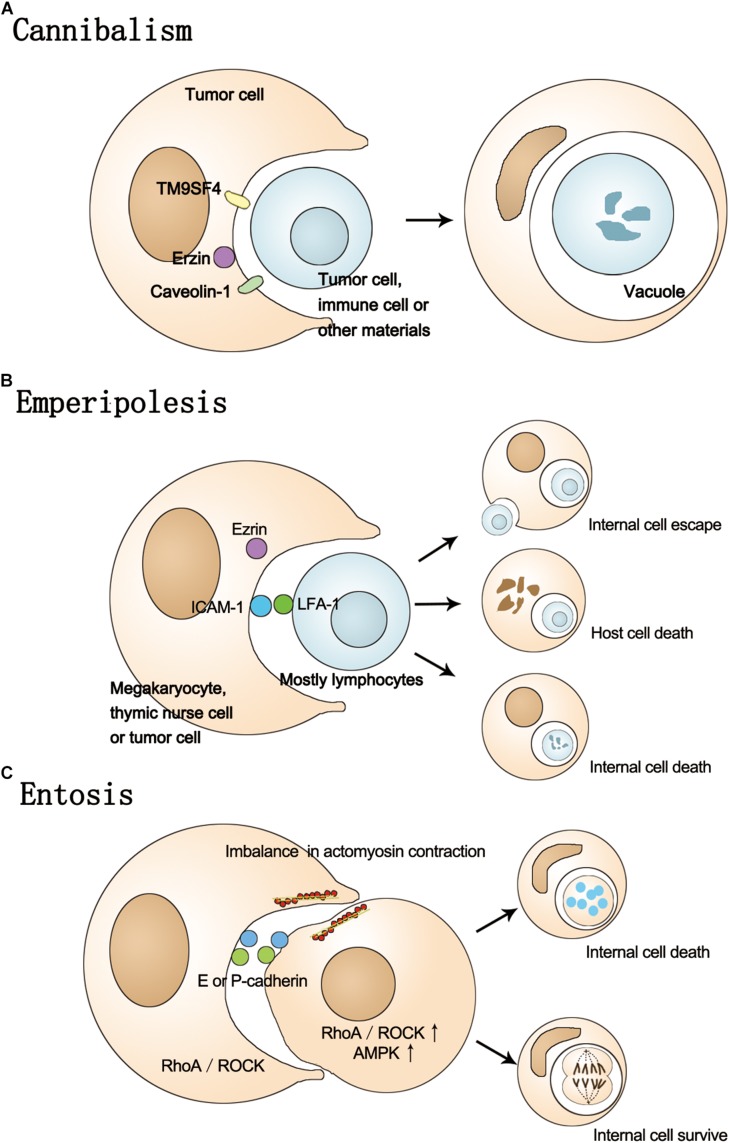
The process of cannibalism, Emperipolesis, and Entosis. **(A)** In the process of cannibalism, host cells actively engulf themselves or other cells, causing the internal cell death. This process involves TM9SF4, Erzin, and caveolin-1. **(B)** The process of emperipolesis involves the Ezrin, LFA-1, and ICAM-1. The fate of inner cells can be escaping from the host or death and host cells can also be destroyed by internal cells. **(C)** Entosis is a homotypic cell-in-cell phenomenon involving E/P-cadherin, Rho-ROCK-actin/myosin pathway and imbalance in actomyosin contraction. The ROCK and AMPK activity is higher in internal cells. The fate of internal cell can be death or survival.

In the process of culturing tumor cells, it is often possible to find some large cells with internal vacuoles and multiple cell nuclei, so-called cell-in-cell structures. Cell-in-cell is a morphological concept (Fais and Overholtzer, 2018) referring to the case where one cell is within another cell, forming a structure like a bird’s eye (Bauchwitz, 1981) or a signet ring (Saphir, 1951). The cells involved can be homotypic or heterotypic, tumor or non-tumor. Although this phenomenon was first observed over 100 years ago, its mechanisms have been studied in more detail in recent years (He et al., 2013). There are several terms that are used to describe this phenomenon, such as cannibalism, emperipolesis, and entosis. In this review, we will introduce these terms and their molecular mechanisms in detail (Figure 1) and discuss the role of the cell-in-cell phenomenon in tumor progression, including the relationship between cell-in-cell structure and the tumor microenvironment (TME), and changes in cellular characteristics.

## Cannibalism

Cannibalism (from canı’bal in Spanish, in connection with alleged cannibalism among the Caribs), also called anthropophagy (from the Greek anthropos “man” and phagein “to consume”) was first used to refer to the act of humans eating other humans (Fais, 2007). A similar phenomenon occurs among cells. The earliest records of cannibalism at a cellular level date back 100 years (Steinhaus, 1891). Such cell structures are characterized by a crescent-moon-shaped morphology, as the nucleus of the host cell is pushed by the large vacuole containing the ingested cells (Yang and Li, 2012). This phenomenon appears in a variety of tumors and is considered a symbol of malignancy (Lugini et al., 2006; Malorni et al., 2007; Matarrese et al., 2008).

The cell engulfed by the host can be a homotypic cell, as often occurs in tumors. For example, in breast cancer, this homotypic cannibalism can be a useful indicator for the diagnosis of squamous cell carcinomas of the breast (Kinoshita et al., 2018). It also occurs between heterotypic cells such as leukocytes and tumor cells. For instance, in a study of 500 cases of oral squamous cell carcinoma, neutrophil-tumor cell cannibalism was observed in 1.4% of cases (Fu et al., 2017). There is another form of cannibalism in which one malignant cell engulfs another, and this complex is further engulfed by another cell. Alternatively, one cell may engulf two cells at once. This is called “complex cannibalism” (Sarode et al., 2012). In melanoma, this phenomenon is observed only in metastatic cells, not those derived from primary tumors, suggesting that there is some correlation between cannibalism and progression of tumors. Cannibalism may also be a mechanism of tumor immune escape owing to “eating” of immune cells (Huber et al., 2005; Caruso et al., 2012).

The cannibalism process is associated with phagocytosis, a common phenomenon among cells with an important role in the resolution of inflammation, antigen presentation, and disposal of apoptotic cells (Lim et al., 2017). The cells responsible for phagocytosis can be divided into two main types: professional phagocytes, such as macrophages; and non-professional phagocytes, such as epithelial cells (Han et al., 2016). Macrophages can be further divided into anti-inflammatory and pro-inflammatory cells according to their surface receptors. The former promote tumor development by helping with immune system evasion, whereas the latter can inhibit tumors via phagocytosis of tumor cells (Yuan et al., 2015). These observations suggest a relationship between phagocytosis and tumor progression that is similar to cannibalism. However, there are important differences between cannibalism and phagocytosis. First, the purpose of engulfment differs in the two cases. The purpose of cannibalism is to provide nutrients (Lugini et al., 2006; Lozupone and Fais, 2015), whereas that of phagocytosis is mainly elimination (Lim et al., 2017). Second, cannibal cells prefer to engulf live sibling cells, whereas macrophages exclusively phagocytose dead cells (Sharma and Dey, 2011). When metastatic melanoma cells were co-cultured with live lymphocytes, they were found to cannibalize them at high rates, which allowed the metastatic cells to survive even under serum-starved conditions; by contrast, the ingestion of latex beads did not promote survival and the cancer cells died quickly (Lugini et al., 2006).

Research has identified several factors that induce cannibalism, including a lack of nutrition (Sharma and Dey, 2011). It is well known that autophagy occurs under conditions of insufficient nutrition, whereas tumor cells may show cannibalism, similar to the way that *Bacillus subtilis* bacteria eat siblings when their carbon source is limited (Hofler et al., 2016). Another factor is increased acidity (Lugini et al., 2006; Fais, 2007). Tumor cells undergo glycolysis even under aerobic conditions, owing to the Warburg effect (Otto, 2016); this causes an accumulation of lactic acid in the TME, and the resulting decrease in pH activates cannibalism-associated enzymes (Lozupone and Fais, 2015). Regional acidosis also plays an important part in tumor metastasis and increasing drug resistance (Fais et al., 2014; Sonehara et al., 2019), which may be related to cannibalism.

## Molecular Mechanism of Cannibalism

The molecular mechanism of cannibalism involves caveolins, ezrin, and TM9. Caveolins are the major structural proteins of caveolae, comprising caveolin-1 (Cav-1), Cav-2, and Cav-3. Cav-1 and Cav-2 promote tumor metastasis (Fu et al., 2017). The endolysosomal compartment of cannibal cells contains large amounts of Cav-1, suggesting that it participates in the cannibalism process (Fais, 2007). Ezrin is a general cross-linker between cortical actin filaments and plasma membranes. It regulates cytoskeletal organization by integrating rho guanosine 5′-triphosphatase (GTPase) signaling (Kawaguchi et al., 2017) and is expressed on phagocytic vacuoles of melanoma cells, which are involved in cannibalism (Lugini et al., 2003). Ezrin also contributes to the connection between actin and caveolin-1-enriched vacuoles of tumor cells, which form the driving structure of the cannibalistic process (Lugini et al., 2006). Altering this connection through various agents can inhibit cannibalism (Fais, 2007). TM9 is a nine-transmembrane-segment protein belonging to a highly conserved family of proteins. It may have key roles in phagocytosis, adhesion, and nutrient sensing (Fais and Fauvarque, 2012). TM9SF4, a member of the TM9 superfamily (TM9SF) in humans, is overexpressed in metastatic melanoma cells but undetectable in cells of primary lesions. TM9SF4 knockdown inhibits the cannibalism phenomenon (Lozupone et al., 2009). TM9SF4 can also regulate autophagy; it localizes to lysosomes and has been shown to regulate autophagy initiation in response to nutrient starvation by inhibiting the nutrient-sensing kinase complex mammalian target of rapamycin complex 1 (mTORC1), and it knockdown inhibits the autophagy (Sun et al., 2018). TM9SF4 is thought to suppress both cannibalism and autophagy, indicating a relationship between autophagy and cannibalism. Studies have also shown that TM9SF4 can bind to the ATP6V1H subunit of the proton pump to active V-ATPase, which regulates the pH gradient in tumor cells (Lozupone et al., 2015); increased acidity in the microenvironment is considered to be an inducer of cannibalism. The fate of the engulfed cell is usually apoptotic cell death (He et al., 2013; Kale, 2015).

## Emperipolesis

Emperipolesis is derived from the Greek (em-inside; peri-around; polemai-wander about). It was first described 50 years ago as the active penetration of one cell by another, which remains intact (Humble et al., 1956). It has been proposed that cell-in-cell and emperipolesis should be used as general terms to refer to cell-in-cell structures or the cell movements associated with them, whereas entosis, cannibalism, and cytophagocytosis should be used to refer more specifically to particular mechanisms of cell-in-cell formation (Overholtzer and Brugge, 2008).

Emperipolesis is a heterotypic cell-in-cell phenomenon that mainly involves histiocytes and megakaryocytes but has also been observed in tumor cells (Xia et al., 2008), for instance, neutrophil cells engulfed by megakaryocytes in the bone marrow (Yener and Dikmenli, 2011) and thymocytes engulfed by thymic nurse cells in the thymic cortex (Overholtzer and Brugge, 2008; Guyden et al., 2015). Thymic nurse cells were first identified in mice in 1980 (Wekerle et al., 1980). They are epithelial cells in the thymus that may contain up to 200 thymic lymphocytes and express both class I and class II MHC complexes on their cell membrane. Thymic nurse cells play an important part in thymocyte development by forming heterotypic cell-in-cell interactions, that is, emperipolesis (Guyden et al., 2015). Rosai–Dorfmann disease is a histiocytic proliferative disorder, in which emperipolesis can be observed in lymph nodes with mixed inflammatory infiltrate and in the cerebrospinal fluid (Kraeft et al., 2008; Cohen Aubart et al., 2018; Piris et al., 2018). Emperipolesis has also been observed in renal cell carcinoma (Rotterova et al., 2018), squamous cell carcinoma (Tetikkurt et al., 2018), and other cancers, as well as in infectious liver diseases (Hu et al., 2015). Natural killer (NK) cells can induce liver fibrosis remission by killing hepatic stellate cells (HSCs). NK cells from liver cirrhosis patients were shown to enter HSCs, impairing the anti-fibrosis capacity of the NK cells through TGF-β-dependent emperipolesis (Shi et al., 2017). The fate of engulfed cells is different from that in cannibalism, as an engulfed cell can escape from the host (Larsen, 1970) or undergo mitosis (Wekerle et al., 1980; Xia et al., 2008). As immune cells and tumor cells are in competition for their respective survival, the co-survival of these two cells is similar to the phenomenon of commensalism. The biological significance and mechanisms of emperipolesis require further investigation (Chen et al., 2013). It only involves living cells, whereas cannibalism can make use of dead or dying cells and other materials in the extracellular space (Gupta et al., 2017).

The engulfed cell can be destroyed and, according to its death mode, there are different terms to describe this procedure. For instance, the CD8+T cells within hepatocytes undergo non-apoptotic death, and are considered to actively invade the hepatocytes rather than being engulfed. This process is called suicidal emperipolesis (Benseler et al., 2011; Sierro et al., 2015). Granzyme B is an enzyme that exists mainly in NK cells and cytotoxic T lymphocytes and can induce apoptosis (Hlongwane et al., 2018). The granzyme B released from the inner NK cell is trapped in vacuoles, which in turn cause apoptosis of the NK cells. This process, called emperitosis (combining emperipolesis and apoptosis), describes how cytotoxic lymphocytes in tumor cells undergo death by apoptosis, enabling immune escape of the tumor cells (Wang et al., 2013). The host cell may also be destroyed; killing of tumor cells containing lymphocytes has been observed (Wang et al., 2009; Takeuchi et al., 2010). Emperipolesis is thought to be a pathway by which NK cell-mediated tumor cell death is regulated (Xia et al., 2008). When the cell-in-cell phenomenon occurs in tumor cells, emperipolesis may be prioritized over entosis, which will be discussed below. The priority given to the formation of this heterotypic cell-in-cell structure suggests that tumor cells can modulate their microenvironment by unique mechanisms (Chen et al., 2013).

## Molecular Mechanism of Emperipolesis

Normal transcellular migration activities and the receptors that regulate them are considered to be important for emperipolesis (Overholtzer and Brugge, 2008). These include extracellular free calcium and adhesion molecules, and the actin-based cytoskeleton and ezrin (Xia et al., 2008). Emperipolesis was shown to be reduced by inhibition of actin polymerization (Takeuchi et al., 2010). The abnormal P-selectin located on the demarcation membrane system of neutrophils and megakaryocytes is associated with their contraction and has been proposed as the cause of emperipolesis in marrow fibrosis (Centurione et al., 2004). Lymphocyte function-associated antigen-1 (LFA-1 or CD11a/CD18) can mediate intercellular interactions between leukocytes and non-blood cells. Together with its ligand, intercellular adhesion molecules 1 (ICAM-1/CD54) (Reina and Espel, 2017), it is thought to be associated with emperipolesis, which can be blocked by co-administration of an anti-LFA1 antibody *in vivo* (Overholtzer and Brugge, 2008). HOZOT is a novel human Treg cell line established from umbilical cord blood mononuclear cells. HOZOT cells can actively penetrate into target cancer cells and form cell-in-cell structures, in a process categorized as emperipolesis. MHC class I seems to be the primary molecule used by HOZOT cells to recognize target cells, and ICAM-1 is one of the molecules recognized by HOZOT cells. In addition, anti-CD62L causes partial inhibition of the cell-in-cell activity of HOZOT cells (Takeuchi et al., 2010).

## Entosis

Compared with cannibalism and emperipolesis, entosis is a relatively new cell-in-cell concept proposed in recent years. The name is from the Greek word “entos,” meaning “inside or within.” First observed in human mammary epithelia, it is a phenomenon of homogenous living cells that occurs in epithelial and other human tumors, involving the invasion of one live cell into another, followed by the degradation of internalized cells by lysosomal enzymes (Overholtzer et al., 2007).

It is well known that cells undergo apoptosis when unattached to the extracellular matrix (ECM) or adhering to inappropriate locations, in a process called anoikis (Paoli et al., 2013). Anoikis probably acts as a barrier to tumor formation or metastasis. However, inhibition of anoikis is not sufficient for long-term survival of tumor cells detached away from the ECM. Entosis has been shown to compensate for apoptotic defects in detached cell populations (Overholtzer et al., 2007). Matrix de-adhesion appears to induce entosis (Ishikawa et al., 2015), and indeed the phenomenon is observed mostly in suspended samples, for example, fluid exudates, urine, and bile (Xia et al., 2008). However, recent research has shown that entosis can also occur when cells are attached to the matrix (Wan et al., 2012; Durgan et al., 2017; Garanina et al., 2017). Mitosis is a inducer of entosis in matrix-attached conditions. A study has shown that entosis occurs in human bronchial epithelial cells in adherent culture. Tracking these cells, the researchers found that the engulfed cells were undergoing mitosis or had done so recently. Inhibition of mitosis led to a profound decrease in cell-in-cell formation in matrix-attached but not in detached cells, leading to the conclusion that mitosis may drive entosis in adherent cell populations. Thus, matrix detachment and mitosis are factors inducing entosis. Anchorage independence and unrestrained proliferation are two classic features of cancer (Durgan et al., 2017). Entosis is induced by nutrient starvation, especially glucose withdrawal. The withdrawal of glucose from growth medium was shown to induce a high level of entosis, and re-addition of glucose to the medium inhibited this process. Interestingly, more inner cell death occurred under the glucose-starved condition than in the full media (Hamann et al., 2017).

Entosis is thought to promote competition between tumor cells, providing nutrition to winner cells from engulfed loser cells in the absence of nutrition. This facilitates the proliferation and metastasis of tumor cells (Sun et al., 2014b). The fate of entotic cells is usually death, but they may also escape from the host and then divide. A small percentage of cells undergo cell division while still internalized, revealing that the engulfed cell is still alive (Overholtzer et al., 2007). Entotic cell death is executed non-cell-autonomously by the autophagy-pathway-dependent lysosomal degradation of live engulfed cells (Krishna and Overholtzer, 2016). Cell death occurs in the absence of caspase-3 cleavage, a hallmark of apoptosis, and dying entotic cells do not exhibit morphological features of apoptosis such as nuclear condensation and fragmentations. Apoptosis, autophagy, and necrosis are well-known forms of cell death (Kroemer et al., 2009). It has been suggested that entotic cell death should be defined as a new type IV cell death (Martins et al., 2017). The fate of entotic cells shows that entosis can inhibit the progression of a tumor by killing matrix-detached tumor cells. However, recent research indicates that entosis may also promote tumor progression by inducing changes in cell ploidy (Krajcovic et al., 2011). Entosis directly promotes polyploidy by disrupting cell division, leading to the formation of binucleate engulfing cells in culture (Krajcovic and Overholtzer, 2012).

## Molecular Mechanism of Entosis

As discussed, the matrix induces entosis. After matrix detachment, the establishment of adherence among cells is an essential step in formation of cell-in-cell structures (Durgan and Florey, 2018). E-cadherin and P-cadherin are key components of the adherent junctions of cells (Vieira and Paredes, 2015; Mendonsa et al., 2018). Cell-in-cell structures cannot form in tumor cells with insufficient E- and P-cadherin, but they occur when E- or P-cadherin is re-expressed (Sun et al., 2014a). Another study showed that alpha-catenin is also related to cell-in-cell formation (Wang et al., 2015). After adherence among cells, entosis is driven by an imbalance in actomyosin contraction between neighboring cells (Gupta et al., 2017). This process is mediated by the small GTPase RhoA and its effector kinase rho-kinase (ROCK I/II) (Overholtzer et al., 2007; Sun et al., 2014a). Cell-in-cell structure formation is blocked when these are inhibited (Durgan and Florey, 2018). As discussed above, mitosis drives entosis in adherent cell populations. This process is regulated by CDC42, a protein with a role in the attachment of cells to each other and to the ECM. CDC42 has been shown to increase mitotic de-adhesion and rounding. This may be because CDC42 constrains mitotic RhoA activation in polarized epithelial cells, and its loss causes RhoA overactivation during metaphase (Durgan et al., 2017).

Bcl2 is a member of the anti-apoptotic B cell lymphoma 2 (BCL2) family, which are key players in the regulation of intrinsic apoptosis (Cui and Placzek, 2018). Caspase-3, which is found at a convergence of the intrinsic and extrinsic apoptotic pathways, is the main executioner of apoptosis (Lossi et al., 2018). Overexpression of Bcl2 or treatment of cells with a caspase inhibitor has no effect on cell internalization. Although entosis is a non-apoptotic procedure, Bcl2 can partially inhibit non-apoptotic cell death by entosis, possibly via an alternate apoptotic program causing cell death when lysosome function is inhibited (Overholtzer et al., 2007). Once ingested, internalized entotic cells are surrounded by a vacuole. Maturation of this vacuole involves modification by autophagy pathway proteins that direct lipidation of the autophagosomal microtubule-associated protein 1 light chain 3 (LC3) on the entotic vacuole, followed by lysosome fusion and internalized cell death and degradation. Knockdown of Atg5 (a protein associated with autophagy) in hosts but not internalized cells can inhibit LC3 recruitment and partially rescue internalized cells from death, demonstrating that autophagy lipidation machinery in host cells causes internal cell death (Florey et al., 2011). Autophagy is a lysosomal delivery pathway, which mediates the formation of double-membrane autophagosomes that wrap cellular components for delivery to the lysosome (Yang and Klionsky, 2010a). These autophagosomes are formed in part by conjugation of LC3 to phosphatidylethanolamine (Yang and Klionsky, 2010b). Entotic cell death requires the LC3 lipidation machinery, which forms the core of autophagy. However, the autophagy pre-initiation kinase complex, composed of Ulk1/2 kinase and the Fip200 and Atg13 adaptor proteins, is not required. This suggests that the LC3 lipidation on the entotic vacuole is different from that in typical autophagy (Florey et al., 2011; Florey and Overholtzer, 2012).

Unlike cells engulfed by phagocytes, the engulfed cell plays an active part in this process, which is thus more like cell invasion than engulfment. RhoA activity and phosphorylated myosin light chain 2 (pMLC2), actin, myosins (MHC IIA and IIB), and ROCK I/II in internalizing cells are higher than in host cells. Overexpression of RhoA or ROCK I/II is sufficient to drive the uptake of epithelial cells (Sun et al., 2014a). Recent research has shown that glucose starvation can induce entosis by increasing the activity of AMP-activated protein kinase (AMPK), as in the case of cannibalism. When cells are short of glucose, AMPK activity increases. Higher AMPK activity occurs in the internal cell than in the host cell, decreasing the deformability of the inner cell. AMPK activity is considered to affect loser cell behavior by regulating cell deformability (Hamann et al., 2017).

## Cell-In-Cell Phenomenon and Tumor Microenvironment

In 1889, Paget’s “seed and soil” hypothesis laid the foundation for the concept of the TME. He thought of the tumor cells as seeds and the distal organs as soil, and proposed that they interact with each other (Paget, 1889). Now, the term TME refers to the entirety of the components of a solid tumor, comprising its vasculature, tumor-associated fibroblasts, various immune cells, and the ECM (Nandigama et al., 2018). The TME has recently become a hot topic in oncology. However, its precise definition is still disputed. Some researchers divide it into six layers with distinct mechanisms and therapeutic approaches. Whether the TME supports the development of the tumor or the tumor changes it to favor its development is controversial (Laplane et al., 2018). The TME contains stromal cells, fibroblasts, and immune cells, which can be induced by tumor cells to produce large amounts of growth factors and cytokines. These factors can induce entosis (Fais and Overholtzer, 2018).

Recently, the relationship between inflammation and cancer has been the subject of increasing attention. It has been suggested that inflammation promotes tumor progression via epithelial-to-mesenchymal transition (Ahn et al., 2018). Interleukin-8 (IL-8) is one of the factors that regulate inflammatory response. A study showed that entosis was inhibited by IL-8 knockdown and significantly enhanced by treatment with recombinant IL-8. This is related to the regulation of intercellular adhesion and the expression of adhesion molecules by up regulation of P-cadherin. IL-8, an inflammatory cytokine, may also recruit neutrophils to regulate entosis (Ruan et al., 2018). Another study found that ulcerative colitis and colon cancer showed more cell-in-cell phenomena than normal colon tissue, colon polyps, and adenomas. Following treatment of colon cancer tissue with high levels of the inflammatory mediator Interleukin-6 (IL-6), the formation of cell-in-cell structures increased compared with tissues exposed to low levels of IL-6. These cell-in-cell structures were formed mostly between tumor cells and cytotoxic T cells, via emperitosis. IL-6 in the TME was also shown to promote the formation of cell-in-cell structures by upregulating the expression of cell adhesion molecule ICAM1, as well as increasing the motility of inner CTL8 cells through the signal transducers and activators of transcription (STAT)3/5, extracellular signal-related kinase (ERK), and Rho-ROCK signaling pathways (Wang et al., 2019). These results suggest a possible interplay between cell-in-cell structures and the TME.

Another important characteristic of the TME is extracellular acidosis. Owing in part to the Warburg effect, the continuous production of acidic metabolites and the resulting enhanced acidity of the outside of the cell lead to consistent acidification of the TME. Thus, the formation of an antacid tumor cell population is promoted, and its invasive and metastatic potential is increased (Bohme and Bosserhoff, 2016). Low pH is among the factors inducing cannibalism. Cannibal cells are particularly resistant to low pH, and can survive acidic conditions under which macrophages and other cells often die (De Milito and Fais, 2005). Extracellular acidosis can activate some lytic enzymes involved in cannibal activity, including cathepsins and other proteases. Thus, extracellular acidosis is considered to have a key role in increasing and maintaining cannibal behavior in tumor cells (Lozupone and Fais, 2015).

Besides nutrient starvation and extracellular acidosis, there are other factors involved in the cell-in-cell phenomenon. The p53 protein is a product of the cancer suppressor gene TP53. Its functions include regulation of the cell cycle, aging, apoptosis, repair of DNA damage caused by genotoxic agents, angiogenesis, and regulation of oxidative stress (Foulkes, 2007). Mutation of the TP53 gene has been shown to increase entosis, possibly via a process associated with epidermal growth factor receptor (EGFR) and integrin expression. Interestingly, glucose starvation did not induce entosis among mutant p53 cells (Mackay et al., 2018). Other tumor suppressor genes such as cyclin dependent kinase inhibitor 2A (CDKN2A) and oncogenes such as Kras and c-Myc have also been shown to be related to the formation of cell-in-cell structures (Mackay and Muller, 2019). A study of cell competition in entosis found that a cell with mutant oncogenic KrasV12 emerged as the winner cell. In general, non-tumorigenic cells are engulfed during entosis, but mutant KrasV12 enables them to become winner cells, possibly owing to the mutant Kras activating Ras-related C3 botulinum toxin substrate 1 (Rac1), resulting in actomyosin contraction inhibition and an increase in the mechanical deformability of non-tumorigenic cells (Sun et al., 2014b).

## Cell-In-Cell and Changes in Related Cellular Characteristics

In cell-in-cell processes, both the host cells and those that are engulfed but escape may undergo changes in their biological functions or acquire new characteristics (He et al., 2013). The biggest change in host cell characteristics is the number of chromosomes. Chromosomes are constantly shuffling as they divide, and the genome has a high rate of change during this process. This is termed chromosomal instability (CIN) (Johnson and McClelland, 2019), and often leads to abnormal chromosome structure and aneuploidy. CIN is also thought to promote tumor evolution and metastasis (Bakhoum et al., 2018). Research has shown that entosis can cause aneuploidy in host cells, owing to the internal cell physically blocking the mitosis of the host cell such that CIN occurs (Krajcovic et al., 2011).

T cells originating from bone marrow do not express the surface markers CD4, CD8, or αβ-T cell receptor (αβTCR) before entering thymic nurse cells. After release from the cell-in-cell structure formed with thymic cells, they acquire different surface markers and have different biological functions (Guyden et al., 2015). In a cell-in-cell selection model, some internalized cells were shown to escape with new characteristics, while others were killed in the vacuole (He et al., 2013). Megakaryocytes are a type of hematopoietic cell, originating from hematopoietic stem cells. Megakaryocytes undergo a terminal maturation process, including multiple rounds of intranuclear division and cytoplasmic recombination to form platelets after maturity (Noetzli et al., 2019). Research shows that neutrophils and megakaryocytes could form cell-in-cell structures by emperipolesis. This process is mediated by an interaction between neutrophil B2 integrins and megakaryocyte ICAM-1/ezrin. Membrane labeling has shown that this process involves the exchange of cell membranes, including the proteins on the membranes, although the nature of these proteins remains to be determined. Hallmark neutrophil proteins such as CD18 and Ly6G (lymphocyte antigen 6 complex locus G6D) are not detectable on the megakaryocyte membrane. The neutrophils transfer its membranes to the megakaryocyte membrane, and acquires membrane from the megakaryocyte after release. Both host cells and invading cells may undergo changes in function during this process (Cunin et al., 2019).

Mitochondria are organelles that carry genetic information and energy independently and can be transferred between different cells, retaining complete function (Hayakawa et al., 2016). Fluorescence co-localization was used to show that mitochondria could be transferred from lymphocytes to tumor cells through cell-in-cell phenomenon, although they were only retained in the tumor cells for a short time (Wang et al., 2019). To determine whether a similar mechanism exists in tumor cells requires further study. We propose that if they escape from other tumor cells and acquire features of the host such as mitochondria or receptors on the plasma membrane, this would support their survival and metastasis.

## Cell-In-Cell in Tumor Progression and Therapy

Studies support that the significance of cell-in-cell structures for tumor progression is twofold. Such structures can limit tumor progression by promoting the death of internalized cells; by contrast, they can also promote tumor progression by digesting the cells swallowed and screening out tumor cells with a high degree of malignancy through cell competition (Wang et al., 2019). In most instances, however, cell-in-cell structures occur in higher-grade cancers. This indicates that the cell-in-cell phenomenon may overall benefit the tumor and promote its progression (Durgan and Florey, 2018) The cell-in-cell phenomenon represents a form of competition between tumor cells, which may promote tumor progression in two ways: (1) the winning cells have a high failure rate of cytokinesis, resulting in aneuploidy; and (2) winning cells obtain nutrients from the cells they engulf, enabling them to survive and proliferate in the absence of nutrients (Florey et al., 2015). As discussed above, the cell-in-cell phenomenon results in a change in CIN by disturbing mitosis. In addition, the chromosome components of engulfed cells can be easily detected in host cells, suggesting that chromosome exchange occurs between engulfed cells and host cells. Tumor progression caused by inflammatory factors may be due in part to inflammatory factors such as IL-6 and IL-8 promoting the formation of cell-in-cell structures and thus CIN (He et al., 2013).

The two opposing roles of the cell-in-cell phenomenon in tumor progression suggest that treatment strategies should be considered in terms of two aspects, that is, artificially either promoting or inhibiting the formation of cell-in-cell structures. Entosis has been increased using methylseleninic acid in pancreatic cancer, resulting in internal cell death (Miyatake et al., 2018). Conversely, formation of cell-in-cell structures has been inhibited by reducing the acidity of the TME by the use of proton pump inhibitors and buffers such as sodium bicarbonate or citrate (Fais et al., 2014), or by inhibiting AMPK to limit entosis (Finicle et al., 2018). Drug resistance is a problem during cancer treatment. Nintedanib can promote entosis during prostate cancer treatment by modulating the CDC42 pathway, resulting in drug resistance (Liu et al., 2019). Entosis may provide a safe environment where a tumor cell can avoid chemotoxic drug exposure (Mackay and Muller, 2019). Interestingly, one particular case of breast carcinoma displayed extensive lymphocyte emperipolesis. This tumor had been nearly eliminated by chemotherapy before surgery, suggesting that emperipolesis might have enhanced the cytotoxic effects of chemotherapy, perhaps via the release of cytokines (Saxena et al., 2002). Thus, the cell-in-cell phenomenon may regulate tumor sensitivity to chemotherapy drugs. However, these therapy strategies are still at the stage of speculation, and further experimental verification is required.

## Cell-In-Cell Structures in Pancreatic Cancer

As discussed above, the cell-in-cell phenomenon is often thought to promote tumor progression by providing nutrition, allowing cells to evade immune surveillance, increasing polyploidy, and so on. It’s true in the case of pleomorphic giant cell carcinoma of the pancreas (PGC). Giant-cell-containing neoplasms of the pancreas present as three types: osteoclastic, pleomorphic, and mixed (Moore et al., 2009). They are rare pancreatic tumors, which account for less than 1% of all pancreatic malignancies (Loya et al., 2004). The pleomorphic giant cell carcinoma of pancreas refers to the tumor consists of malignant mononucleated and multinucleated giant cells, and the cell-in-cell structures, cannibalism, have been observed in it (Jalloh, 1983; Silverman et al., 1988; Gupta and Wakefield, 1992). The carcinomas with osteoclast-like giant cell in pancreas are first reported several decades ago (Rosai, 1968). It consists of undifferentiated, pleomorphic, mononuclear cells and multinucleated osteoclast-like giant cells (Georgiou et al., 2016). The 2010 World Health Organization has grouped them together as undifferentiated carcinoma with osteoclast-like giant cells (OGC) (Temesgen et al., 2014). Studies show that OGCs have a better prognosis with estimated 5-year survival rate of 59.1% compared to pancreatic ductal adenocarcinoma (PDAC) (Muraki et al., 2016), but PGCs are with early metastasis and poor prognosis similar or worse to PDAC (Temesgen et al., 2014). This suggests that the cannibalism in PGCs may promote the tumor metastasis.

However, there is an exception in PDAC (Cano et al., 2012). Nuclear protein 1 (nupr1), also known as p8, is a potential mediator of PDAC resistance to cell stress (Hamidi et al., 2012). It is highly expressed during the progression of PDAC and other cancers (Ito et al., 2005a, b). Inhibition of nupr1 in PDAC cells resulted in homotypic cell cannibalism, leading to cell death. This was could be enhanced by transforming growth factor β (TGFβ) and reduced the metastasis of PDAC. So there is considered to be an association between cell-in-cell structures and good prognosis. Such homotypic cannibalism is different from entosis in that it is not reduced by inhibiting the expression of ROCK (Cano et al., 2012). However, entosis can also occur in pancreatic cancer cells (Khalkar et al., 2018). Methylseleninic acid (MSeA) was shown to induce cell-in-cell structure formation in pancreatic cancer cells through entosis regulated by CDC42 and CD29, and this process led to death of the inner cell (Miyatake et al., 2018). MSeA has been shown to be a superior anticancer selenium compared with sodium selenite in multiple organ sites, and is expected to be effective in the pancreas as well, as it suppresses growth of pancreatic cancer cell lines (Wang et al., 2014). In conclusion, we propose that cell-in-cell structures in PGCs may cause the tumor progress, while in PDAC may indicate a better prognosis, as these structures can inhibit tumor metastasis by killing the inner tumor cell.

## Conclusion

Cannibalism, entosis, and emperipolesis are the three main processes by which cell-in-cell structures are formed. The relationships among these three concepts require further study. They influence tumor progression in two ways. On the one hand, they promote cancer progression by providing nutrition, helping tumor cells to escape immune surveillance, and increasing genetic instability. On the other hand, they can inhibit tumor growth via cells eating each other, which causes the death of the internalized cells. The cell-in-cell phenomenon in tumors can help us to understand more about the biology of tumor cells and determine the prognosis of tumor patients. Through cell-in-cell processes, both host cells and the cells that are engulfed but escape may undergo changes in their biological functions or acquire new characteristics. This may inspire new approaches for treating malignant tumors, by artificially promoting or inhibiting the formation of cell-in-cell structures. We propose that it may be possible to improve the cell recognition ability of phagocytes to cancer cells via new characteristics acquired by cell membrane exchange. It may also be possible to promote chemotherapy-sensitive tumor cells to engulf insensitive tumor cells by cell-in-cell processes before chemotherapy. Research on cell-in-cell death pathways will increase our knowledge of cell death, as well as its implications for heath and disease. These phenomena involve morphological features that are easily identifiable under a light microscope, making their study more economical and practical than research involving expensive molecular techniques. So far, the three different cell-in-cell phenomena have usually been studied separately. Future research should focus on the relationships among them, for example, the difference between the homotypic and heterotypic cell-in-cell phenomena. In addition, entosis is considered to be a cell death pathway, but its relationship to other cell death pathways such as autophagy and apoptosis requires further study. Finally, cell-in-cell structures seem to have the opposite effects in in PDAC compared with other cancers, but the relationships between these structures and the specific biology of PDAC remain to be explored. Further study that addresses these aspects may lead to new approaches for treating malignant tumors.

## Author Contributions

XW, YL, and BS contributed to the conception and design of the study. HC and HZ searched literature and provided suggestions for revision. XW wrote the first draft of the manuscript. JL, RK, GW, YW, and JH wrote sections of the manuscript. All authors contributed to the manuscript revision, and read and approved the submitted version.

## Conflict of Interest

The authors declare that the research was conducted in the absence of any commercial or financial relationships that could be construed as a potential conflict of interest.
